# Anti-adherence capacity of phytosphingosine on titanium surfaces

**DOI:** 10.1177/08853282251334902

**Published:** 2025-04-20

**Authors:** Enni Liinoja, Nagat Areid, Elisa Närvä, Floris J. Bikker, Vuokko Loimaranta, Timo O. Närhi

**Affiliations:** 1 Department of Prosthetic Dentistry and Stomatognathic Physiology, 8058 University of Turku, Turku, Finland; 2 Institute of Biomedicine and FICAN West Cancer Centre Laboratory, 8058 University of Turku and Turku University Hospital, Turku, Finland; 3 Department of Oral Biochemistry, Academic Centre for Dentistry Amsterdam, 1192 University of Amsterdam and VU University Amsterdam, Amsterdam, the Netherlands; 4 Institute of Dentistry, 8058 University of Turku, Turku, Finland; 5 Wellbeing Services County of South-West, Finland

**Keywords:** Anti-Adherence, antimicrobial, titanium, titanium dioxide, adhesion, fibroblast, phytosphingosine

## Abstract

Firm soft tissue attachment on oral implant components together with good bacterial control are important prerequisites for uneventful implant healing. TiO_2_ coatings have been shown to enhance human gingival fibroblast attachment, but the coating does not have antimicrobial properties. Phytosphingosine (PHS) is known to have antifouling properties against the cariogenic bacterium *Streptococcus mutans (S. mutans)* which is also among the first colonizers on implant surfaces. This makes PHS an interesting agent to prevent microbial adhesion on dental implant surfaces. The aim of this study was to examine the impact of PHS on *S. mutans* and human gingival fibroblast adhesion on titanium surfaces with or without TiO_2_ -coating. Titanium discs (*n* = 99, diameter 14 mm, thickness 1 mm) were fabricated for the study. The discs were divided into four groups: (1) non-coated discs (NC), (2) titanium discs with hydrothermally induced TiO_2_ coatings (HT), (3) NC discs treated with PHS solution and (4) HT discs treated with PHS solution. Hydrophilicity of the discs was evaluated by water contact angle measurement. *S. mutans* was added on HT and NC discs with or without PHS treatment for 30 minutes and the number of attached bacteria was estimated by plate counting method. For fibroblast experiment, the cells were plated on the discs and the number of adhered fibroblasts was determined at three time points (1, 3, 6 h). Additionally, confocal microscope images were obtained to examine fibroblast and *S. mutans* adhesion and to evaluate cell spreading. PHS treatment significantly decreased the hydrophilicity of HT and NC titanium surfaces (*p* < .001). *S. mutans* adhesion was significantly reduced after PHS treatment on both NC (*p* < .001) and HT surfaces (*p* < .001). Fibroblast adhesion was significantly reduced in HT group at 1 and 3h time points (*p* < .001), situation leveling out by the 6th hour. PHS reduced the number of adhered fibroblasts to the surface at incubation times of 1 hours (*p* = .0011) and 3 hours (*p* = .0194). At the 6 hour time point the number of adhered cells was no longer reduced, but still a reduction in cell spreading on the surface was observed (*p* < .05). The adhesion differences were present only in HT group. The PHS treatment reduced adherence *of S. mutans* and fibroblasts on TiO_2_ coated titanium, which may result from reduced hydrophilicity of the surfaces. The dual approach of PHS treatment and TiO_2_ coating could provide microbial antifouling properties of dental implants but may also affect fibroblast adhesion.

## Introduction

Dental implants are constantly exposed to oral bacteria owing to partial contact with the jawbone and the gums, and thus being prone to infections.^1^ The prevalence of postoperative infections after implant placement is 2–3% among patients and two-thirds of the infected implants fail.^2,3^ Therefore, there is a constant need for new antimicrobial applications to prevent oral implant infections. The basic elements of biofilm formation on teeth and dental implant structures are comparable. The protein pellicle adherence on tooth or dental implant surface is first required, which is then followed by adherence and colonization of predominantly Gram-positive streptococci. This allows the formation of multispecies biofilm.^4^

Fibroblast adhesion is crucial for the establishment of a stable peri-implant soft tissue structure, but proper adhesion is also essential in preventing peri-implant infections. Normally, a connective tissue is formed around a bioinert material, such as titanium. It is also possible to improve the attachment of tissues to titanium surface, a good example of which is titanium dioxide (TiO_2_) -coating which mediates direct and tight fibroblast attachment on different surfaces.^5–7^ This is associated with enhanced hydrophilicity and surface structure of TiO_2_ coatings consisting of nearly spherical nanoparticles of 20-50 nm.^6^ Nonetheless TiO_2_-coating itself does not have direct antimicrobial or bacterial adhesion preventing properties which could improve implant healing.

Sphingolipids are components of eukaryotic cell membranes involved in the regulation of cell growth, differentiation and programmed cell death.^8,9^ Sphingolipids may have anti-erosive properties due to their hydrophobicity which may function as a protective barrier for ions diffusing to the underlying surfaces. Phytosphingosine (PHS, 4-hydroxy sphinganine) is a positively charged sphingoid base which has an affinity for negatively charged surfaces such as calcium hydroxyapatite.^10^ The previous studies have shown PHS having antimicrobial properties.^11–13^ For example, PHS on glass surfaces reduced the adhesion of salivary bacteria^14^ and a PHS coating on bare and saliva-coated hydroxyapatite discs had significant anti-adherence capacity against oral bacteria including *Streptococcus mutans*.^12^ Both anti-adherence and bactericidal properties of PHS give indications of its possible use in controlling biofilms. However, the anti-adherence capacity of PHS against *S. mutans* on titanium surfaces has not been demonstrated before. This study aimed to assess the attachment of PHS on noncoated and TiO_2_ -coated titanium surfaces and to evaluate its effect on surface hydrophilicity. Furthermore, the effect of PHS on *S. mutans* adhesion as well as the attachment of human gingival fibroblasts on noncoated and TiO_2_ -coated surfaces were evaluated.

## Materials and methods

### Titanium discs

Titanium discs (diameter 14 mm, thickness 1 mm) were grinded from both sides with silicon carbide paper of 1200 grit. The discs were then cleaned ultrasonically in 100 % acetone, 70 % ethanol and distilled water for 5 minutes. 48 discs were coated with a thin film of nanostructured TiO_2_ using hydrothermal treatment (HT) and 53 discs were noncoated (NC). HT processing employs the exceptional water solubility of nearly all inorganic substances under conditions of heightened temperature and pressure. This technique facilitates the precipitation and crystallization of the dissolved material from the fluid medium.^15^ The HT suspension was created as described earlier.^6,16^ Shortly, titanium dioxide (TiO_2_), purified water, and 1:10 diluted tetramethylammonium hydroxide (TMAH) (N(CH_3_)_4_^+^OH^−^) were mixed for 5 minutes. The hydrothermal suspension was then added to a Teflon container which consisted of a Teflon inner vessel and a stainless-steel jacket with titanium substrates at the bottom. The vessel was kept at a temperature of 150 ± 10°C in an oven for 48 hours. After the 48-h treatment period, the titanium substrates were removed from the vessel and left to cool in the air. Finally, all discs were cleaned in acetone, ethanol, and distilled water, as presented above, and air dried.

### PHS adsorption on titanium discs

To test whether PHS adsorbed on the titanium discs, a stock solution (5 mg/mL) of PHS (Doosan Cooperation, Lui-Dong, South Korea) was made in absolute ethanol. Of this, dilutions were made in 20 mM Tris (pH 6.8), supplemented with 0.1% Tween 20 (Tris-Tween) to obtain PHS concentrations of 0 — 250 µg/ml. Adsorption of PHS on the discs was tested essentially as described.^10^ A total of 10 discs were used and they formed two different treatment groups in total (NC and HT with PHS). Shortly, the titanium discs were incubated with PHS for 60 min in a shaker (37°C, 300 r/min, Eppendorf Thermomixer comfort) after which the discs were washed three times with Tris-Tween and three times with sterilized water to remove unbound sphingolipid. The discs were then transferred to a clean 24-well plate and adsorbed PHS extracted with ethanol. Ethanol with the extracted PHS was transferred to a clean well and the ethanol was allowed to evaporate. The precipitated PHS was suspended in 150 μL of 70% ethanol and 100 μL of this solution was transferred to a 96-well plate with 25 μL of *ortho*-phthalaldehyde reagent (OPA, P0532-Phthaldialdehyde Reagent, Sigma-Aldrich, Burlington, Massachusetts, USA). Fluorescence was measured by (BioTek Synergy HT Microplate Reader, Winooski, Vermont, USA) at 360 nm excitation and 460 nm emission wavelengths. The quantity of adsorbed sphingolipid was determined by reference to a corresponding standard curve, created using sphingolipid concentrations of 0-50 μg/mL. All the measurements were conducted at least in duplicates.

### Contact angle measurement

In this study, contact angle measurement was done to investigate the effect of PHS on the hydrophilicity of NC and HT discs. The discs were treated with PHS (100 µg/ml) or buffer (controls) as described above. A total of 12 discs were used and they formed four different treatment groups in total (NC with and without PHS, HT with and without PHS).

Sessile contact angle measurement was done visually with a contact angle meter (Attension 7 ® Theta, Biolin Scientific AB, Finland). A droplet (3-4 μl) was deposited on the surface and a high-resolution camera captured the image from the profile. The contact angle was then measured by image analysis software (OneAttension, Nanoscience Instruments, Phoenix, USA). Contact angle means were then calculated from the data. The contact angle was measured from at least three droplets on both sides of the disc.

### Surface roughness measurements

The average surface roughness (Sa) of NC, HT, NC-PHS, and NC-PHS specimens were measured using a 3D non-contact optical profilometer (Bruker Nano GmbH, Billerica, MA, USA). A total of six measurements per group were evaluated on randomly selected points with a 5x objective lens and a 0.5 multiplier, using a back scan and length parameters of 20 μm and 60 μm in VSI/VXI mode to obtain a 3D image of the specimen surfaces. Software (Vision 64) was used to analyze surface areas and roughness parameters.

### Bacterial adhesion assay

For the bacterial adhesion test, NC and HT discs were treated with 100 μg/mL PHS-solution as described above. A concentration of 100 μg/mL was selected because higher concentrations did not result in a significant increase in PHS adsorption to the surface. 24 discs were used, six in each group (NC, NC-PHS, HT, HT-PHS).

*Streptococcus mutans* (NCTC 10,449) was grown overnight in brain-heart-infusion (BHI, BD Bacto^TM^, USA) medium. The bacteria were collected by centrifugation (5000*g*, 10 min) and washed once with PBS (diluted from Dulbecco’s Phosphate-Buffered Saline, D-PBS 10x, Invitrogen). The cells were resuspended in PBS to OD_550_ = 0,35. The adhesion was done as described earlier^17^ with some modifications. Briefly, PHS coated and non-coated titanium discs were rolled in cell suspension for 30 min after which the discs were washed three times with PBS to remove unbound bacteria. The attached bacteria were carefully scraped from the surfaces using small applicator brushes (Quick-Stick, Dentsolv AB, Sweden) and the detached bacteria as well as the applicators were applied into transport media, tryptic soy broth (TSB). Two applicators were used to scrape the cells from both sides of the disc, four applicators in total.

The samples were vortexed and serial dilutions (1:10, 1:100, 1:1000) were used for plate cultivation. Plates were grown anaerobically for 4 days at 37°C and the number of bacterial cells was counted as colony forming units (CFU). CFU was determined by square millimeter to ensure that small differences in disc size did not affect the obtained results. For the microscopy analysis, *S. mutans* bacteria were allowed to adhere to discs as described above. The attached bacteria were then stained with Carboxyfluorescein diacetate, succinimidyl ester (CFDA-SE) (2 μM in PBS buffer supplemented with 1 mM EDTA and 0.01% Tween) for 60 minutes at 37°C. After staining, the bacteria were washed with PBS and fixed with 4% paraformaldehyde (PFA) for 2 hours at 4°C. Following fixation, the samples were washed twice with PBS and stored in fresh PBS at 4°C, protected from light. A spinning disc confocal microscope (63× Zeiss Plan-Apochromat, 3i CSU-W1 Spinning Disk) was used for cell imaging.

### Fibroblast adhesion assay

NC and HT discs were treated with PHS as described above. Human gingival fibroblasts were used in the cell culture experiment. Human gingival fibroblasts used in this study were obtained, with informed consent, from individuals undergoing tooth extractions at the Institute of Dentistry, University of Turku as described earlier.^18^ The cells were grown in DMEM supplemented with 10% fetal bovine serum and 100 U/μg penicillin-streptomycin (Gibco, BRL, Life Technologies, Paisley, UK) and incubated at 37°C in a 5% CO_2_ environment. Fibroblasts were plated at a density of 25,000 cells/cm^2^ on the titanium discs, five discs in each group (NC, NC-PHS, HT, HT-PHS). After 1, 3 and 6 h of incubation, the discs were washed three times with PBS to remove nonadherent cells. The discs were placed into TE buffer (10 mmol/l Tris-HCl, 1 mmol/l EDTA, pH 7,5) and stored at −70°C. Quantitative DNA measurements were then used to determine the amounts of adhered cells. The thawed samples were sonicated on an ice water bath for 10 min to release genomic DNA. Intercalating dye (PicoGreen© dsDNA quantitation kit, Molecular Probes Europe, Leiden, The Netherlands) was added to the discs, and fluorescence was measured using excitation and emission wavelengths of 490 nm and 535 nm. The amount of DNA was read from a phage dsDNA standard curve.

To analyze the cell morphology and spreading, cells were fixed after 6 h of cultivation using 4% PFA for 15 min at 37°C. The fixed samples were washed with PBS and stored at +4°C. To permeabilize cells discs were treated with 0.5% TRITON-X-100 in PBS for 15 min and washed three times with PBS. Nucleus was stained using DAPI (nucleus staining, 1:200) and Actin structures with Phalloidin Atto-647 (1:400, Sigma-Aldrich, USA) diluted with 30% horse serum in PBS for 1 h protected from light at room temperature. Finally, discs were washed with PBS and glued onto microscope glass using Mowiol (Sigma-Aldrich). A spinning disc confocal microscope (63× Zeiss Plan-Apochromat, 3i CSU-W1 Spinning Disk) was used for cell imaging. Three biological replicates were performed for the confocal microscope. To evaluate cell spreading, the area of individual cells (*n* = 30 per group) was measured with ImageJ and Fiji software based on the actin staining (bottom layer of the confocal image).

### Statistical analysis

The statistical analyses were conducted with JMP Pro version 17. ANOVA (post-hoc Tukey) test was used to compare continuous variables among the groups while the data was normally distributed. The comparison was done with the Wilcoxon Rank Sum Test or Kruskal-Wallis test while when the distribution did not follow normal distribution. Continuous variables are shown as mean ± SD or median (25^th^ and 75^th^ percentile). Confocal microscope images were analyzed with ImageJ, Fiji-program. GraphPad Prism program was used to analyze the data and design the graphs. Differences were considered significant at a 95% confidence level and the alpha value for significance was set at *p* < .05.

## Results

**Figure 1.**
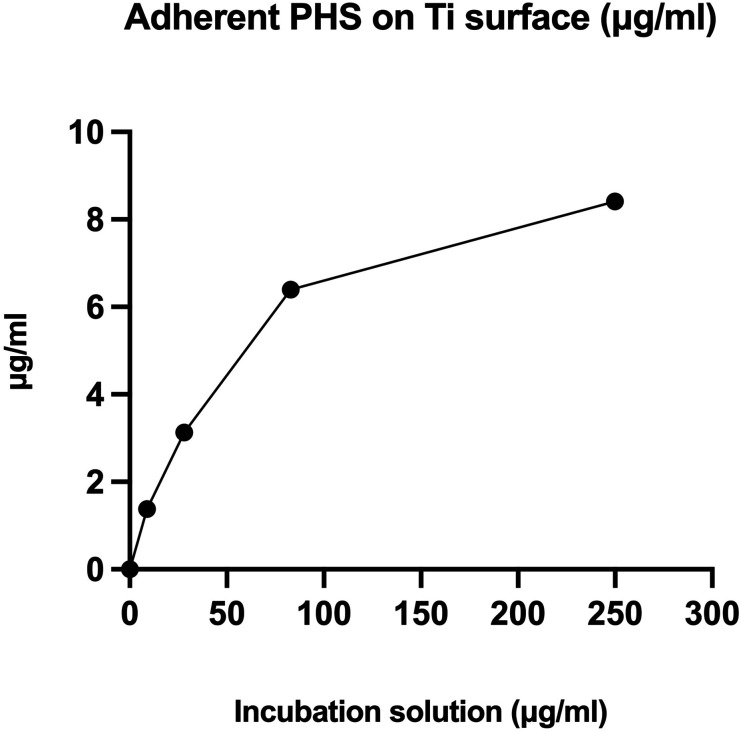
Amount of PHS adhered on NC titanium discs. The amount of adherent PHS was tested on noncoated (NC) titanium discs using the PHS solutions of 0, 9, 28, 83 and 250 µg/ml, *n* = 2.

The PHS appeared to adhere to NC discs in a dose-dependent manner (Figure 1). Based on the results obtained for NC discs, 100 µg/ml was selected for further coating tests.

**Figure 2.**
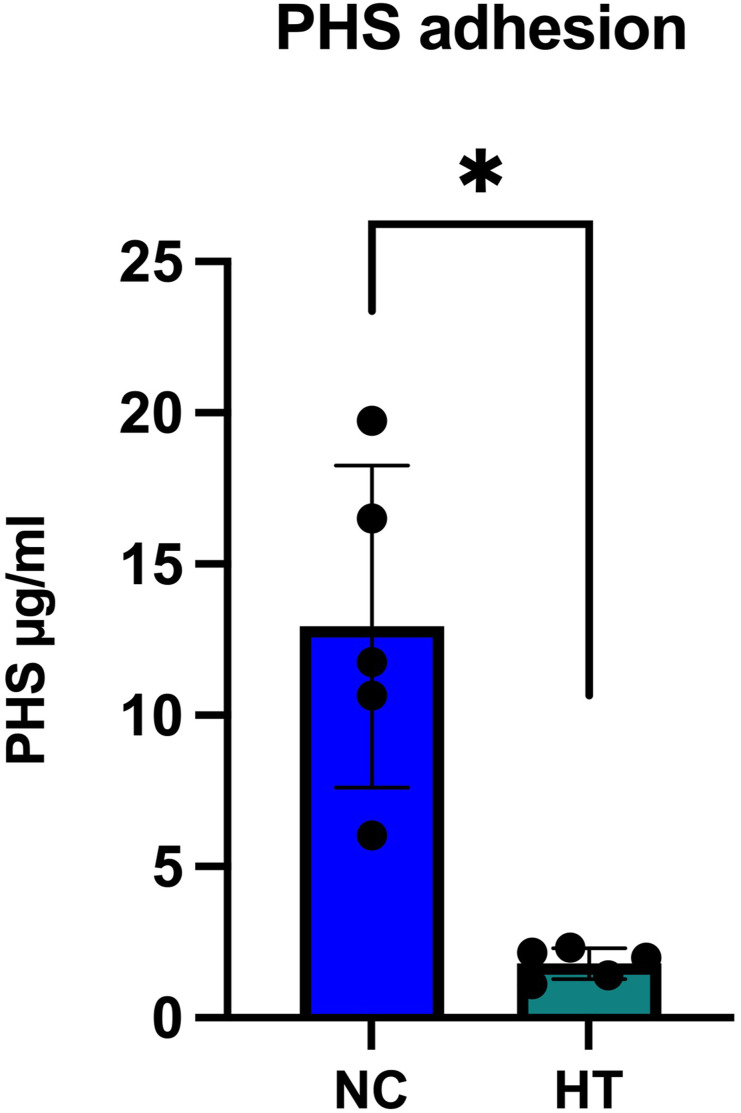
Amount of adhered PHS on HT and NC titanium discs. PHS adhesion was tested on noncoated (NC) and Hydrothermal coated (HT) titanium surfaces using 100 µg/ml PHS. Mean ± SD + individual values, ANOVA. Asterisk indicates statistical significance (*p < .05).

NC and HT discs were treated with 100 µg/ml of PHS. Significantly (*p* = .0122; Wilcoxon rank sum test) more PHS adhered to NC discs (12.9 ± 5.3 µg/ml) than to HT discs (1.8 ± 0.5 µg/ml) (Figure 2).

**Figure 3.**
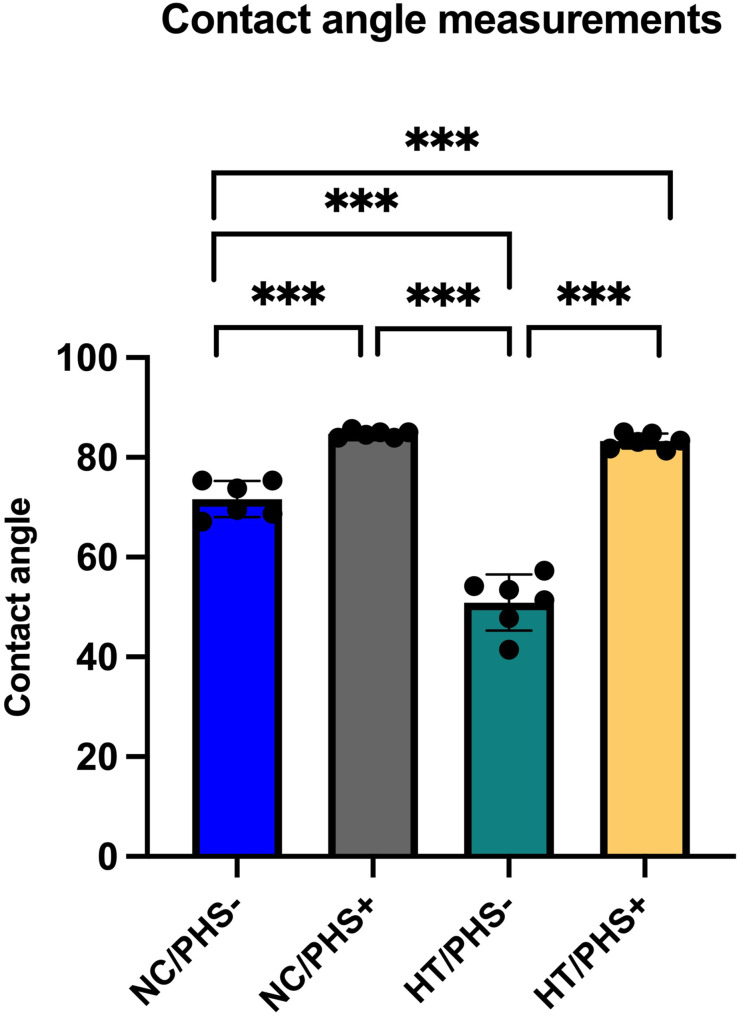
Water contact angle values on NC/PHS-, NC/PHS+, HT/PHS-, and HT/PHS + discs. Mean ± SD + individual values, ANOVA. Asterisk indicates statistical significance (***p < .001).

Without PHS treatment the contact angle of HT discs was significantly lower than that of NC discs (52.38 ± 5.60 vs 71.65 ± 4.67, *p* = .0024, Figure 3). In both groups, contact angles increased significantly after PHS treatment (*p* = .0122; Wilcoxon rank sum test), and reached similar levels (HT: 83.13 ± 4.21 vs NC: 85.05 ± 2.80, *p* = .165).

**Figure 4.**
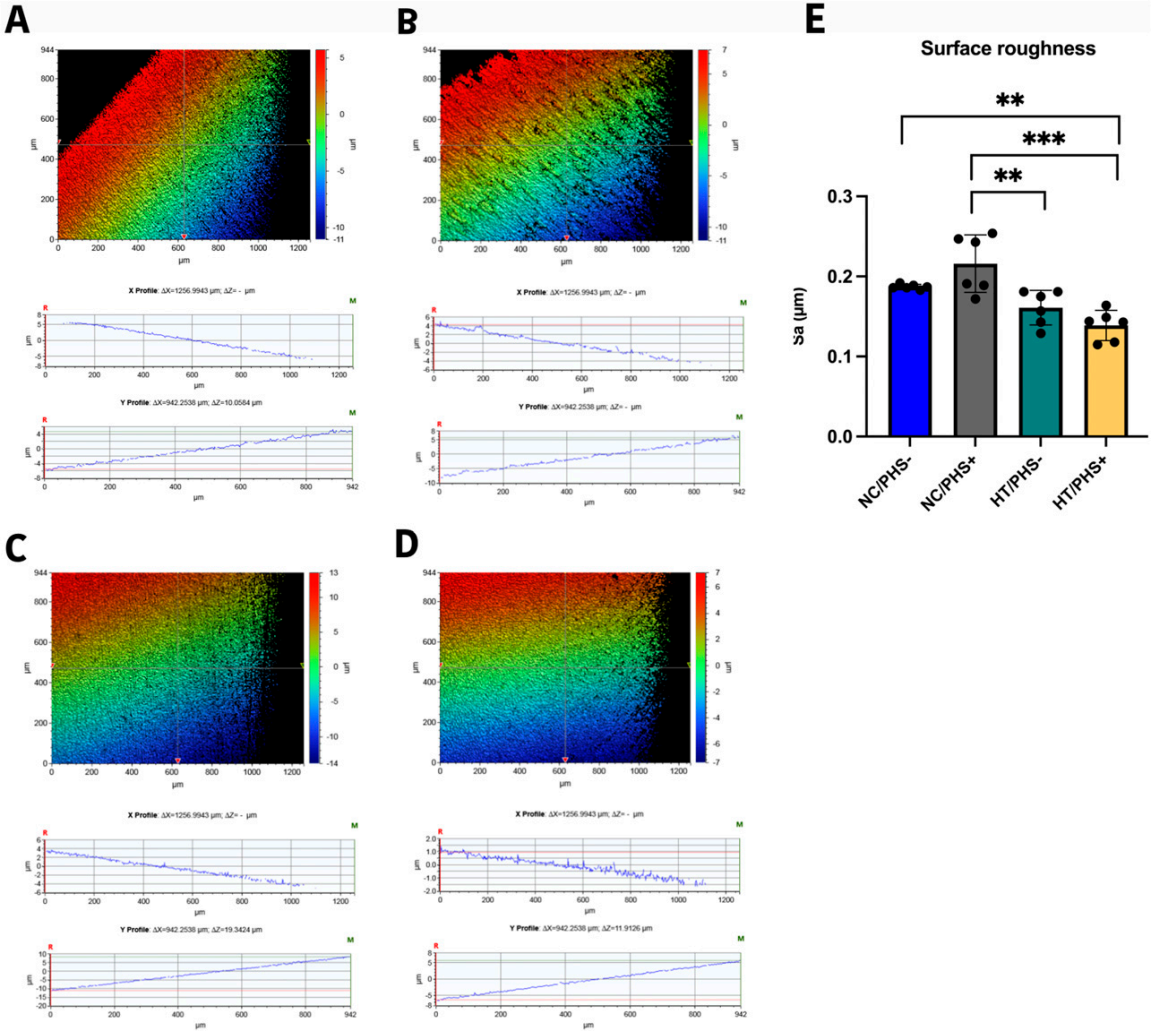
Typical surface profiles (Sa) of NC/PHS- (A), NC/PHS+ (B), HT/PHS- (C), and HT/PHS+ (D) discs. The images were acquired with a 5x objective lens and utilizing a field of view multiplier of 0.5x. (E) Shown Surface roughness values (Sa) of the investigated substrates. Mean ± SD + individual values, ANOVA. (**p < .01, ***p < .001).

To determine, whether the PHS treatment would affect the surface topography, the surface roughness measurements were accomplished. Figure 4 displays the surface roughness profile (Sa) of NC/PHS- (A), NC/PHS+ (B), HT/PHS- (C), and HT/PHS+ (D) discs. The NC/PHS + treated surfaces demonstrated significantly higher surface roughness (0.22 µm) compared to the HT surfaces with and without PHS treatment (*p* < .001, *p* < .01), respectively. However, the HT/PHS + surfaces demonstrated significantly lower surface roughness (0.14 µm) compared to NC surfaces with and without PHS treatment (*p* < .001, *p* < .01), respectively (Figure 4). There was no significant differences within the same group with and without PHS treatment.

**Figure 5.**
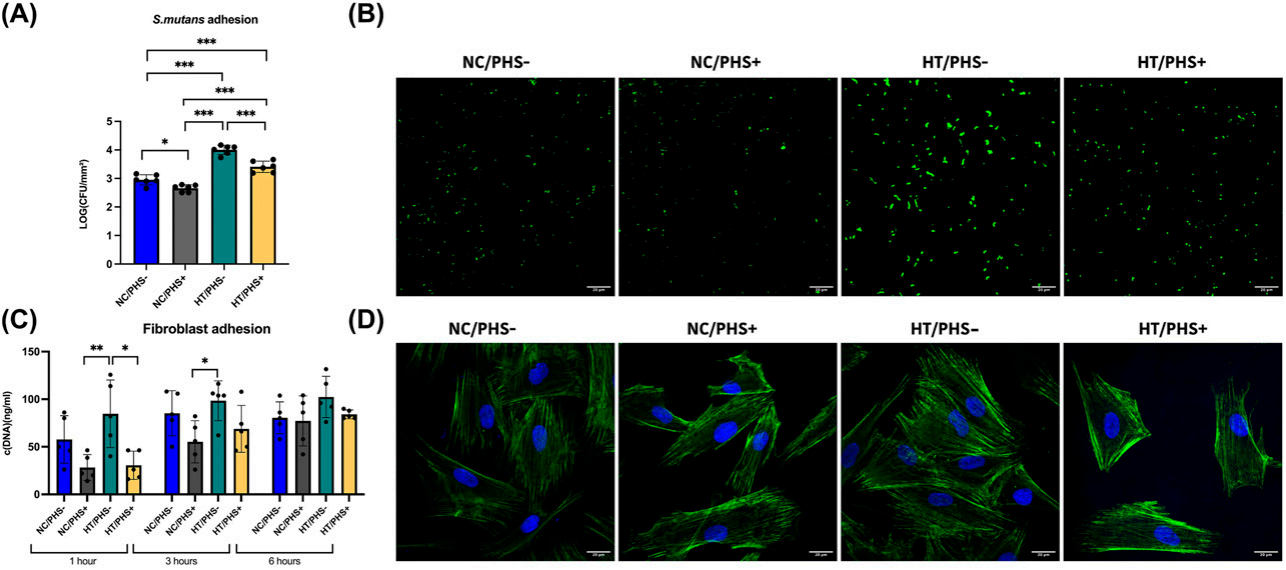
Adherence of *S. mutans* and gingival fibroblasts on NC/HT titanium discs with and without PHS treatment. Representative confocal microscopy images of adherent *S. mutans* on investigated substrates stained with (CFDA-SE) (A). Amount of adhered *S. mutans* (LOG (CFU/mm^2^)) on NC/HT titanium discs with and without PHS treatment (B). Mean ± SD + individual values. Representative confocal microscopy images of the attached gingival fibroblasts on titanium discs after 6 hours, F-actin (green), DAPI (blue), bottom layer of the confocal image, scale bar = 20 μm (C). Quantification of adhered fibroblast DNA on non-coated (NC) and TiO_2_ -coated (HT) titanium discs without and with PHS treatment at each time point (D). Mean ± SD + individual values. ANOVA. (*p < .05, **p < .01, ***p < .001).

After PHS treatment, *S. mutans* adhesion was significantly reduced on both NC (*p* < .001) and HT discs (*p* < .001) (Figures 5(A) and (B)). In pairwise comparisons, a significant difference in *S. mutans* adhesion was observed between NC/PHS+ and NC/PHS- groups with a *p*-value of 0.032 and between HT/PHS+ and HT/PHS- groups (*p* < .001). On HT discs the mean bacterial adhesion was with PHS coating 3.41 ± 0.20 LOG (CFU/mm^2^) and without PHS coating 4.00 ± 0.17 LOG (CFU/mm^2^). On NC discs with PHS coating bacterial adhesion was 2.66 ± 0.12 LOG (CFU/mm^2^) and without PHS coating 2.95 ± 0.18 LOG (CFU/mm^2^).

Due to the fact that PHS treatment reduced surface hydrophilicity while reducing the surface roughness on both NC and HT discs raised the question if this will affect on fibroblast attachment and spreading. Cell culture experiment revealed that PHS treatment reduced initial cell attachment of the fibroblasts in all groups (1 hour, *p* = .0011 and 3 hours, *p* = .0194) (Figure 5(C)). Importantly, fibroblast adhesion increased over time during incubation, and the observed difference was not detected after 6 hours (*p* = .58) (Figure 5(C)), implying that PHS treatment affects negatively only the initial adhesion kinetics. To verify equal cell attachment of the gingival fibroblast on discs, we imaged the actin cytoskeleton after 6 hours. Microscopy imaging revealed normal cell spreading in all conditions (Figure 5(D)).

**Figure 6.**
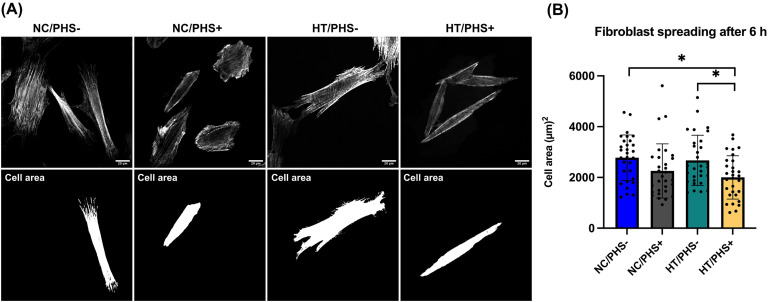
Cell spreading of the gingival fibroblasts on NC/HT titanium surface with and without PHS treatment. Representative images of the actin cytoskeleton and cell area after 6 h (A). Quantifications of cell area after 6 h on NC/PHS-, NC/PHS+, HT/PHS-, and HT/PHS+ (B). (*n* = 30 technical replicates, mean ± SD. Significant*p*values (*p* < .05) are marked in the figures.

To assess cell spreading quantitatively, the approximate areas of attached gingival fibroblasts were measured from confocal microscope images. Cell spreading was analyzed based on actin staining. After 6 h of cell culture, fibroblast cell spreading was decreased on PHS-treated surfaces, reaching significance on HT surfaces (*p* < .05) (Figure 6).

## Discussion

Previous studies have shown that PHS has antifouling properties^11–14^ offering potential benefits in terms of biofilm control on implant surfaces. In this study we showed that PHS treatment of titanium discs has antifouling properties, but PHS may also reduce the initial attachment of fibroblasts on titanium surface.

The ability of PHS to adhere to the HT derived TiO_2_ coated surface was weaker compared to the NC substrate, yet PHS may still sufficiently prevent bacterial growth when used on coated dental implants. Earlier studies^6^ have shown that TiO_2_ coating enhances hydrophilic properties of titanium which may reduce the ability of PHS to adhere to titanium surface. Previously PHS adherence has been shown to be efficient (3.8 µg/cm^2^) on hydroxyapatite discs.^10^ Hydroxyapatite is the main constituent of dental enamel. In this study PHS adherence on NC titanium surface was significantly less efficient (0.26 µg/cm^2^), but it still retained antifouling properties. TiO_2_ coating further reduced PHS adherence (0.036 µg/cm^2^). Previous studies have demonstrated that chemical treatment of titanium surface can enhance surface functionality and adhere antimicrobial agents to the surface.^19^ Similar implementations could possibly improve the adherence of PHS to the surface. Despite PHS adhering less effectively to the titanium surface, it seems to adhere sufficiently well to reduce *S. mutans* adhesion.

When interstitial fluids are exposed to artificial materials, their interaction is influenced by the wettability of the material.^20^ This interaction is characterized by the adsorption of proteins, attachment of cells, and the effect of water and they determine the extent of cell adhesion, cell differentiation, and the eventual formation of tissue at the interface.^21^ The contact angle is a crucial parameter that provides information about the wettability of a solid surface. Hydrophilic properties of titanium decreased during PHS treatment, contact angle being higher on PHS + discs compared to PHS- discs on both NC and HT surfaces. This behavior might be explained by the way the charged phosphate part of PHS sticks to the charged titanium surface, exposing the hydrophobic tail. Statistical significance in contact angle differences between the NC and HT groups after PHS treatment could not be discerned in the present study either parametrically or non-parametrically. This suggests that PHS treatment leads to a similar contact angle outcome regardless of whether the surface is NC or HT, potentially due to its ability to modify surface wettability.

Additional factors such as surface topography, roughness, and chemistry, play a vital role in the initial cell response at the cell-material interface, ultimately influencing soft tissue health and stability.^22^ All examined substrates demonstrated a surface roughness of 0.2 µm or less-a threshold below which surface roughness has no significant impact on biofilm formation or colonization.^23^ However, surface chemistry and nanotopography can also change the implant surface interaction with ions, proteins, and cells.^24^ These interactions can positively influence molecular and cellular activities and promote tissue healing at the titanium–tissue interface.^25^

As our results indicate, PHS functions as an anti-adhesive on titanium surfaces. This is in line with earlier studies where PHS treatment was shown to reduce bacterial adherence on hydroxyapatite and glass surfaces.^14^ PHS decreased the hydrophilic properties of the titanium surface, but whether this is the mechanism of the antifouling effect remains to be studied. In earlier studies PHS is shown to have direct antimicrobial properties, for example, by disrupting microbial cell membrane.^13^

The finding that PHS treatment decreased fibroblast attachment on both NC and HT surfaces is in line with previous studies which have shown that salivary coating may interfere with the adherence of fibroblasts on titanium surfaces.^26^ PHS reduced fibroblast attachment at early time points but the difference evens out when the cells have time to adhere to the surface and no difference is seen in number of attached cells after 6 hours. Still, a reduced cell spreading was evident even after 6 hour incubation which may reflect the reduced hydrophilic properties of PHS treated surfaces. Reduced fibroblast adhesion at early time points corresponds to a trend observed in earlier studies, where the adhesion of a young biofilm decreased due to PHS for 3 h but no longer for 6 h.^14^ Investigating alternative concentrations of PHS may facilitate achieving more optimal balance between bacterial and fibroblast binding. However, HT coating enhances fibroblast adhesion^5–7^ which may improve fibroblast adhesion on titanium surface also in the presence of PHS. Since fibroblast adhesion increases on HT + PHS surfaces over time, this combination could potentially be employed in the future for the management of implant biofilms and soft tissue attachment during the early stages of healing.

In our study, combining HT derived TiO_2_ coating and PHS treatment reduced the adherence of *S. mutans* and fibroblasts. Naturally, reduced fibroblast adhesion is an undesired effect, but it remains unclear whether the antifouling properties induced by combined PHS-treatment and TiO_2_ coating offer a greater benefit at the early stage of healing than the reduced fibroblast adhesion and the implications of this phenomenon on ultimate implant wound healing. However, it needs to be taken into consideration that the methods used in this study do not mimic *in situ* situation. The conditions in the oral cavity are fundamentally more complex and further (clinical) studies are needed.

## Resources and collaboration

This study was conducted at University of Turku, Institute of Dentistry, Department of Prosthetic Dentistry and Stomatognathic Physiology in collaboration with the Department of Oral Biochemistry, Academic Centre for Dentistry Amsterdam. The Cell Imaging and Cytometry core facility (Turku Bioscience Center, University of Turku and Åbo Akademi University and Biocenter Finland), EuroBioimaging node in Turku, are acknowledged for services, instrumentation, and expertise.

## Data Availability

Data is available on request from the authors.*
